# Molecular Landscape of Bladder Cancer: Key Genes, Transcription Factors, and Drug Interactions

**DOI:** 10.3390/ijms252010997

**Published:** 2024-10-12

**Authors:** Danishuddin, Md Azizul Haque, Shawez Khan, Jong-Joo Kim, Khurshid Ahmad

**Affiliations:** 1 Department of Biotechnology, Yeungnam University, Gyeongsan 38541, Republic of Korea; danish23@yu.ac.kr (D.); azizul@ynu.ac.kr (M.A.H.); 2 National Center for Cancer Immune Therapy (CCIT-DK), Department of Oncology, Copenhagen University Hospital, 2730 Herlev, Denmark; shawez.jmi@gmail.com; 3 Department of Health Informatics, College of Applied Medical Sciences, Qassim University, Buraydah 51452, Saudi Arabia

**Keywords:** bladder cancer, transcription factors, DEGs, biomarkers

## Abstract

Bladder cancer is among the most prevalent tumors in the urinary system and is known for its high malignancy. Although traditional diagnostic and treatment methods are established, recent research has focused on understanding the molecular mechanisms underlying bladder cancer. The primary objective of this study is to identify novel diagnostic markers and discover more effective targeted therapies for bladder cancer. This study identified differentially expressed genes (DEGs) between bladder cancer tissues and adjacent normal tissues using data from The Cancer Genome Atlas (TCGA). Gene Ontology (GO) and Kyoto Encyclopedia of Genes and Genomes (KEGG) enrichment analyses were conducted to explore the functional roles of these genes. A protein–protein interaction (PPI) network was also constructed to identify and analyze hub genes within this network. Gene set variation analysis (GSVA) was conducted to investigate the involvement of these genes in various biological processes and pathways. Ten key genes were found to be significantly associated with bladder cancer: *IL6*, *CCNA2*, *CCNB1*, *CDK1*, *PLK1*, *TOP2A*, *AURKA*, *AURKB*, *FOXM1*, and *CALML5*. GSVA analyses revealed that these genes are involved in a variety of biological processes and signaling pathways, including coagulation, UV-response-down, apoptosis, Notch signaling, and Wnt/beta-catenin signaling. The diagnostic relevance of these genes was validated through ROC curve analysis. Additionally, potential therapeutic drug interactions with these key genes were identified. This study provides valuable insights into key genes and their roles in bladder cancer. The identified genes and their interactions with therapeutic drugs could serve as potential biomarkers, presenting new opportunities for enhancing the diagnosis and prognosis of bladder cancer.

## 1. Introduction

Bladder cancer is the second most common type of cancer affecting the genitourinary system, and its prevalence is rising worldwide [1,2,3]. The global impact of bladder cancer is becoming increasingly significant, as demonstrated by the rising mortality rate. Therefore, it is critical to improve diagnostic and treatment methods to more effectively combat the disease [4]. In the United States alone, approximately 83,000 new cases of bladder cancer were diagnosed in 2021. Of these, around 17,000 were projected to result in death, underscoring the severity of the disease [5]. The diagnosis and treatment of bladder cancer are generally classified by clinical stage into two main types: low-risk non-muscle invasive bladder cancer (NMIBC) and the more aggressive muscle-invasive bladder cancer (MIBC) [6].

Furthermore, the risk of bladder cancer is not evenly distributed between genders. It is 3.7 times more likely to occur in men than in women. Among non-smokers, men face an even higher age-standardized risk of bladder cancer—4.3 times greater than that of women [7]. The median age at diagnosis also differs slightly by gender, with men typically being diagnosed at 69 years of age and women at 71 years. These statistics highlight the importance of developing effective screening and early detection methods, especially for older populations. Urothelial cell carcinoma (UCC) is the most common form of bladder cancer, accounting for 90–95% of all cases. The treatment of bladder cancer is notably expensive, making it the most costly cancer to manage. This high cost is due to its recurrent nature, which necessitates ongoing disease monitoring, and expensive trimodal therapies. These therapies include surgery, chemotherapy, and radiotherapy, all of which contribute to the financial burden of treating bladder cancer [8].

Advances in molecular diagnostics have significantly transformed the management of bladder cancer, particularly through the discovery of biomarkers that facilitate early detection and provide prognostic information [9,10,11,12]. Firstly, recent research has increasingly emphasized the role of genetic mutations and abnormal gene expression in the development and progression of bladder cancer [13,14,15]. Secondly, the advent of high-throughput technologies, combined with sophisticated computational analyses, has made it possible to identify DEGs that are critical to the molecular pathogenesis of bladder cancer [16].

These studies have not only illuminated the complex gene networks and pathways driving tumorigenesis but have also pinpointed potential targets for therapeutic intervention. Despite these promising developments, it is important to note that key risk profiles for bladder cancer have been established using genes associated with various symptoms. Currently, large-scale publicly available gene expression datasets offer a valuable resource for identifying potentially reliable biomarkers for bladder cancer patients. To further enhance diagnosis and treatment, efforts are increasingly focused on developing frameworks capable of categorizing patients based on clinical data, molecular markers, and prognostic factors. Nevertheless, there remains a gap in research specifically aimed at the precise identification of prognostic biomarkers related to key hallmark pathways and their associated drugs for bladder cancer [2,17]. Addressing this gap could be crucial for advancing personalized medicine and improving outcomes for bladder cancer patients.

This study employs a thorough computational method to thoroughly examine differentially expressed genes (DEGs) in bladder cancer. The main objective is to identify significant genes and their interactions, which could serve as potential biomarkers or therapeutic targets. To begin, gene expression data for bladder cancer were obtained from the TCGA database [18]. Using the dataset, DEGs were initially screened and identified. Next, we conducted a detailed analysis of the expression patterns of these central genes, focusing on their connections to other genes associated with diseases. We also explored the significance of variations in gene expression specific to bladder cancer. Furthermore, a gene–drug interaction analysis was performed to identify potential therapeutic drug candidates that could target these key regulatory genes. By conducting these sequential analyses, our objective is to enhance the understanding of bladder cancer at the molecular level and ultimately contribute to the development of more effective diagnostic tools and treatment strategies.

## 2. Results

### 2.1. Identification of Key Regulators or Hub Genes

**Figure 1:**
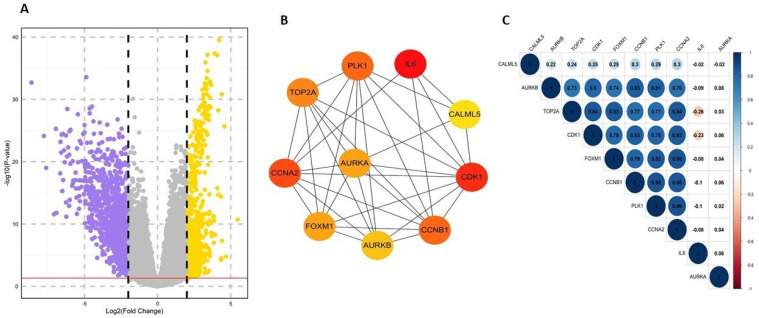
Screening and identification of key genes in the TCGA–BLCA dataset. (**A**) Volcano plots showing differentially expressed genes (DEGs) from the TCGA–BLCA dataset. Upregulated genes are shown in yellow, downregulated genes in violet, and non-significant genes in grey. (**B**) The top 10 hub genes were selected based on degree centrality using the CytoHubba app in Cytoscape. The color indicates the degree of interaction, with more intense red representing higher interaction, while orange and yellow denote intermediate and lower interaction levels, respectively. (**C**) Correlation analysis among the hub genes.

A total of 1539 differentially expressed genes (DEGs) were identified between the bladder and normal samples. Of these, 519 genes were upregulated, while 1020 genes were downregulated. Genes with an adjusted *p*-value < 0.05 and a log_2_-fold change >2 were considered significant. The volcano plot and clustering module of these DEGs are illustrated in Figure 1. To explore the functions and pathways associated with these DEGs, enrichment analysis was performed, which highlighted the roles of these key genes. We examined the topological properties using the Network Analyzer and CytoHubba plugins in Cytoscape. Hubs within the network, characterized by their high degree, are pivotal to the PPI network’s function (Figure 1B). Genes frequently appearing in the top 10 by degree, namely *IL6*, *CCNA2*, *CCNB1*, *CDK1*, *PLK1*, *TOP2A*, *AURKA*, *AURKB*, *FOXM1*, and *CALML5*, were identified as potential biomarkers. Of these, nine genes were upregulated, with *AURKB* showing the highest fold change of 3.56, followed by *TOP2A* with 3.30. Other upregulated genes include *CDK1*, *FOXM1*, *PLK1*, *AURKA*, *CCNA2*, *CCNB1*, and *CALML5*, with fold changes of 3.27, 3.04, 2.91, 2.74, 2.74, 2.57, and 2.88, respectively. In contrast, *IL6* was downregulated, with a fold change of −4.39. The expression levels of these genes were notably higher in bladder cancer patients compared to normal samples. Correlation analyses were conducted to assess the expression relationships among the hub genes. The results revealed a high positive correlation among most of the hub genes, with the exception of *IL6*, *AURKA*, and *CALML5* (Figure 1C).

**Figure 2:**
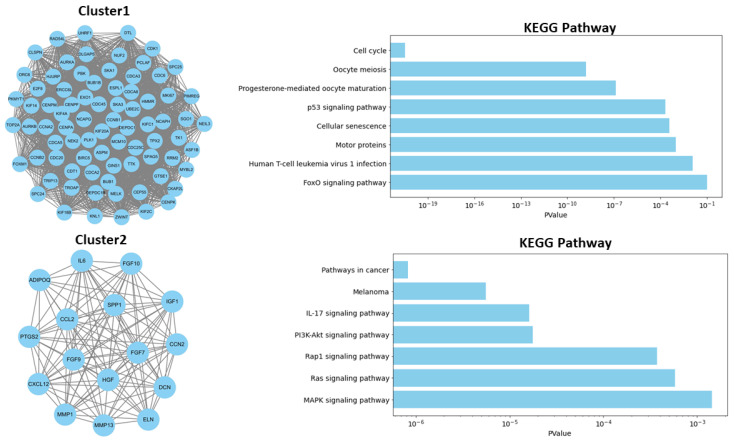
Top modules within the PPI network. These two modules were identified using the MCODE algorithm in Cytoscape with a K-core value of 4, a node score cutoff of 0.3, and a maximum depth of 100, including their interacting gene partners.

To further investigate the associations within the PPI network, the top two modules were extracted using the “MCODE” package in Cytoscape. Cluster 1, which had the highest cluster score, comprised 75 nodes and 2661 edges (Figure 2). In contrast, Cluster 2 consisted of 16 nodes and 111 edges (Figure 2). To elucidate the biological functions of these clusters, we performed functional annotation analysis using the DAVID database for each module separately. The most significantly enriched biological processes and KEGG pathways are summarized in Figure 2. Genes in Cluster 1 were predominantly associated with the cell cycle, oocyte meiosis, progesterone-mediated oocyte maturation, the p53 signaling pathway, and cellular senescence, whereas genes in Cluster 2 were linked to the IL-17 signaling pathway, the PI3K-Akt signaling pathway, the Rap1 signaling pathway, and the Ras signaling pathway.

**Figure 3:**
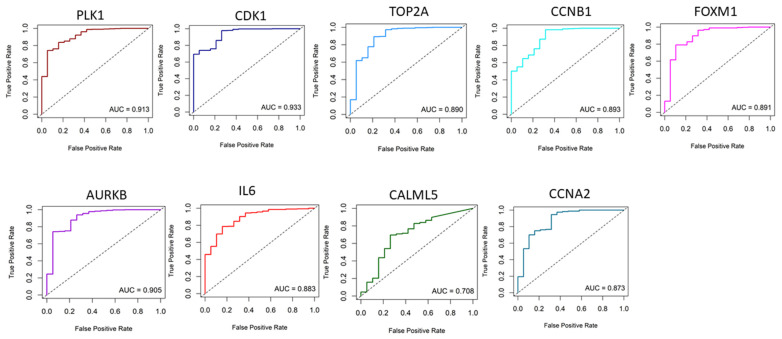
ROC curves for the significant gene expression data. The AUC values suggest that the expression analysis of these markers can effectively differentiate between patient groups with different diseases and controls.

**Figure 4:**
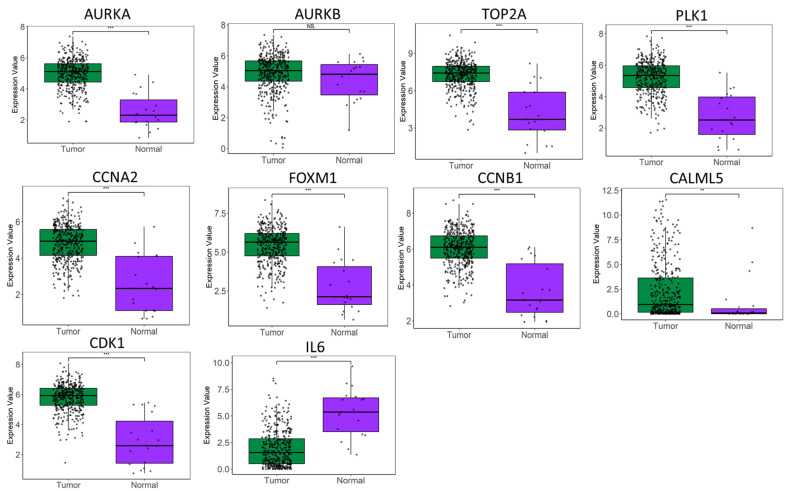
The box plots display the expression levels of several of the ten hub genes that exhibit statistically significant differential expression between normal and bladder cancer samples (** *p* < 0.01; *** *p* < 0.001; NS: Not Significant).

Receiver operating characteristic (ROC) curve analyses further demonstrated the diagnostic and prognostic value of these key genes. The ROC analysis highlighted that the mRNA expression levels of *PLK1*, *CDK1*, *TOP2A*, *AURKA*, *AURKB*, *CCNB1*, *FOXM1*, and *CCNA2* effectively differentiated bladder cancer from normal tissues (Figure 3). Specifically, *CDK1*, with an AUC value of 0.93, followed by *PLK1* at 0.91 and *AURKB* at 0.90, showed the largest areas under the ROC curve, indicating their strong potential for distinguishing between bladder cancer and normal samples (Figure 3). Therefore, after combining the above analysis of the nine hub gene expression levels with the ROC results, we concluded that these genes might be the most potential genes with higher diagnostic values. Except for *IL6*, expressions of other genes were significantly higher in bladder cancer than in the normal sample (Figure 4).

### 2.2. Functional Enrichment Analysis of DEGs

**Figure 5:**
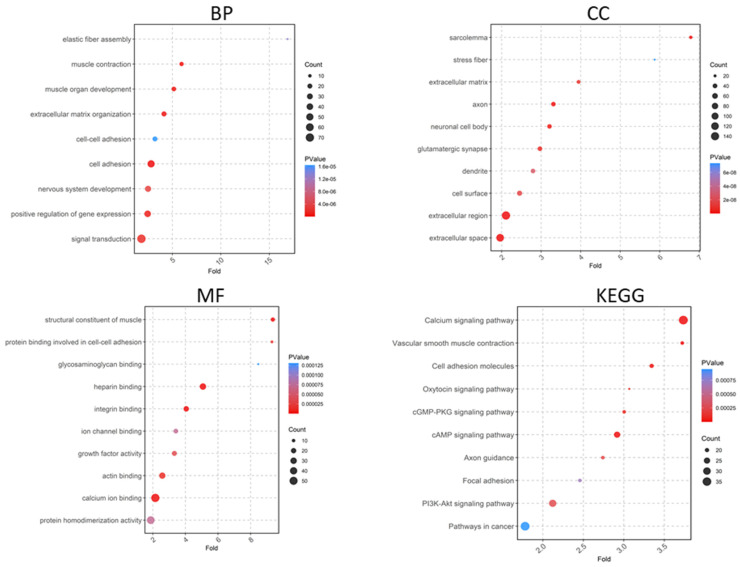
Gene Ontology analyses of upregulated genes include biological process (BP), cellular component (CC), and molecular function (MF), along with Kyoto Encyclopedia of Genes and Genomes (KEGG) pathways.

**Figure 6:**
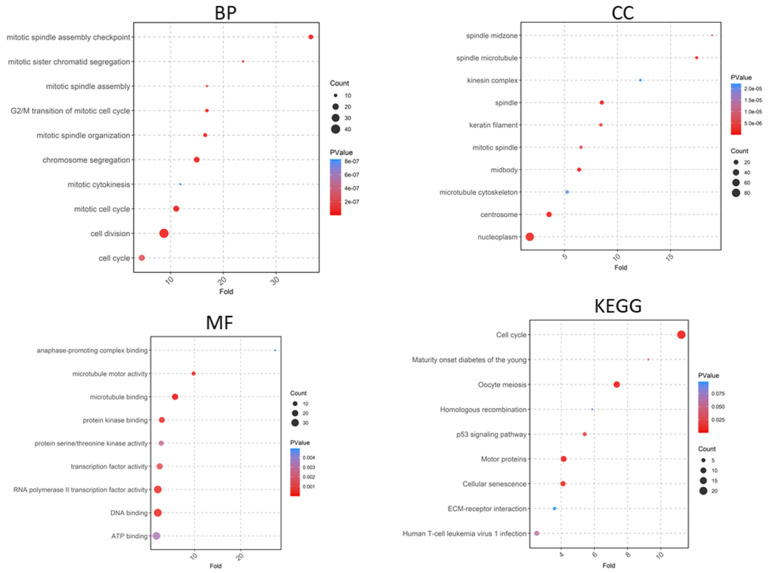
Gene Ontology analyses of downregulated genes include biological process (BP), cellular component (CC), and molecular function (MF), along with Kyoto Encyclopedia of Genes and Genomes (KEGG) pathways.

A total of 519 upregulated and 1020 downregulated genes were analyzed using DAVID software version v2023q4. Gene Ontology (GO) enrichment analysis revealed that upregulated DEGs were associated with biological processes such as muscle contraction, extracellular matrix organization, cell–cell adhesion, and elastic fiber assembly (Figure 5), while downregulated DEGs were linked to cell division, cell cycle progression, and the G2/M transition of the mitotic cell cycle (Figure 6). In the cellular component category, upregulated DEGs were related to the extracellular region, extracellular space, extracellular matrix, and sarcolemma (Figure 5), whereas downregulated DEGs were associated with structures like the spindle midzone, mitotic spindle, keratin filament, and kinesin complex (Figure 6). For molecular function, upregulated DEGs showed enrichment in activities such as extracellular matrix structural constituent, metalloendopeptidase activity, and integrin binding, while downregulated DEGs were related to muscle structural constituents, actin binding, and tropomyosin binding (Figure 5). KEGG pathway analysis identified upregulated pathways, including the calcium signaling pathway, focal adhesion, and the PI3K-Akt signaling pathway (Figure 5), whereas downregulated pathways included the cell cycle, ECM–receptor interaction, and human T-cell leukemia virus 1 infection (Figure 6).

### 2.3. Relationship of Hub Genes and Disease-Related Genes

**Figure 7:**
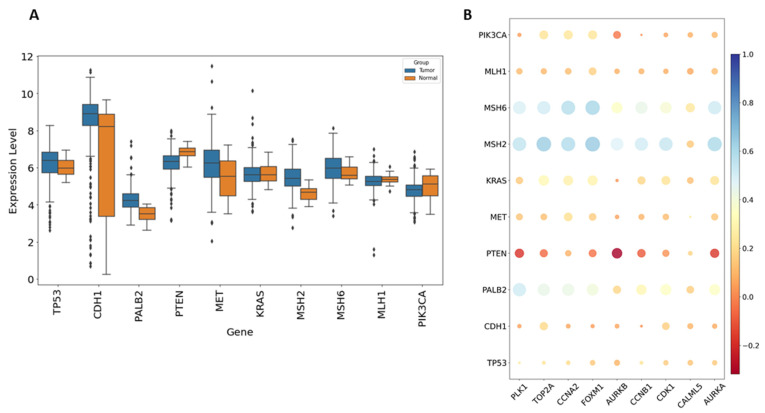
The relationship between hub genes and disease-related genes. (**A**) Comparison of expression levels for various disease-related genes between control samples and individuals with bladder cancer. (**B**) A bubble plot illustrates the Pearson correlations between nine hub genes and disease-related genes.

The disease-associated genes linked to bladder cancer were identified using the GeneCards database (https://www.genecards.org/, accessed on 10 July 2024). GeneCards uses a scoring system to evaluate gene–disease associations, where higher scores indicate stronger evidence of relevance to the specific disease. This analysis revealed notable differences in gene expression levels between control and bladder cancer groups, with significant genes including *TP53*, *MET*, *PTEN*, *PIK3CA*, *CDH1*, *PALB2*, *KRAS*, *MSH2*, *MSH6*, and *MLH1* (Figure 7A). Pearson correlation analysis showed that the expression of these disease-related genes was strongly positively correlated with several hub genes. Specifically, high levels of *CCNA2*, *CCNB1*, *CDK1*, *PLK1*, *TOP2A*, *AURKA*, *AURKB*, *FOXM1*, and *CALML5* were positively associated with *TP53*, *MET*, *PIK3CA*, *CDH1*, *PALB2*, *KRAS*, *MSH2*, *MSH6*, and *MLH1*. Notably, *MSH2* and *MSH6* demonstrated a high correlation with these genes (Figure 7B).

### 2.4. Gene Set Variation Analysis (GSVA) Analyses

**Figure 8:**
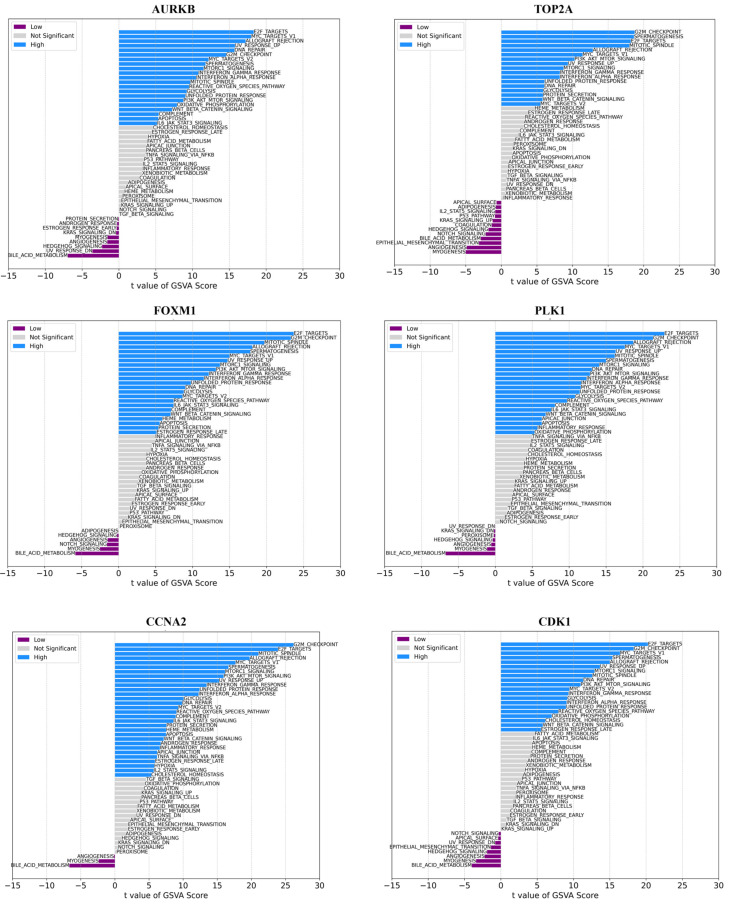

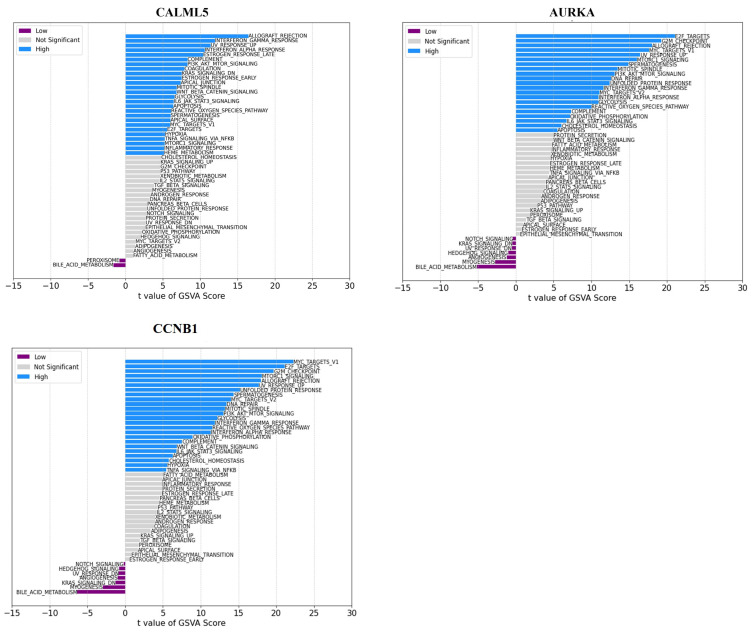
GSVA analysis of high and low expression.

The GSVA methodology transforms expression data into gene set enrichment scores, enabling the evaluation of pathway activity across samples. This allows us to assess how active these pathways are across different samples. We proceeded to analyze the specific signaling pathways involved in the ten hub genes and explored the effect of candidate genes on the signaling pathways related to disease progression. GSVA results showed that high expression of the hub genes primarily enriched E2F targets, PI3-AKT-Motr signaling, mitotic spindle, apoptosis, Wnt/beta-catenin, Apical Junction, and other signaling pathways (Figure 8). Low expression of *AURKB*, *FOXM1*, and *PLK1* genes mainly enriched angiogenesis, bile acid metabolism, and myogenesis pathways, whereas low expression of *TOP2A* enriched in angiogenesis, bile acid metabolism, Notch signaling, coagulation, p53, apical surface, and kras signaling_up pathways (Figure 8).

### 2.5. TF–Gene Regulatory Network Analyses

**Figure 9:**
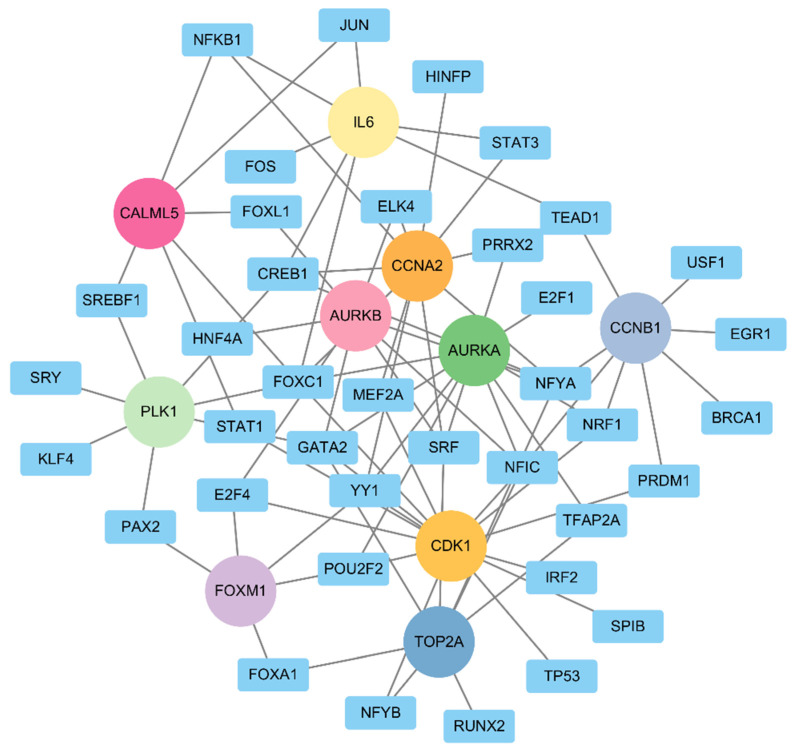
The hub gene–transcription factor (TF) regulatory network. The regulatory network of hub genes and transcription factors (TFs) was obtained using the NetworkAnalyst 3.0 web server. Square nodes represent TFs, and circle nodes stand for hub genes.

For the hub genes we identified, a TF–gene regulatory network was constructed. This network includes 86 interaction pairs among the selected genes and 38 TFs (Figure 9). *CDK1* was found to be regulated by 14 TFs, *AURKA* by 12 TFs, *CCNA2* by 11 TFs, *AURKB* and *TOP2A* by 8 TFs, *PLK1* and *IL6* by 7 TFs, *CALML5* by 6 TFs, and *FOXM1* by 5 TFs. In addition, most of the TFs were found to regulate more than one hub gene. For example, *FOXC1* was identified to connect with *CDK1*, *CCNA2*, *PLK1*, *CALML5*, *AURKA*, and *IL6*. *CREB1*, *GATA2*, *NFYA*, and *SRF* were found to be regulated by four hub genes. Out of 38 TFs, only 12, namely, *E2F1*, *EGR1*, *FOS*, *HINFP*, *HNF4A*, *IRF2*, *KLF4*, *RUNX2*, *SPIB*, *SRY*, and *USF1*, were found to interact with only a single hub gene. These findings indicate that the hub genes might be regulated by these transcription factors and are likely involved in biological processes related to bladder cancer.

### 2.6. Potential Drug Identification

We employed the Drug–Gene Interaction Database (DGIdb) to identify therapeutic drugs; top drugs with reasonable interaction scores were selected. A review of the collected results shows that most drugs are antibodies and inhibitors. Among these selected drugs, Fluorouracil, Genistein, Paclitaxel, Pazopanib, Sorafenim, and Tamoxifen were predicted to target two hub genes (Supplementary Table S1). In summary, these drugs, along with other drugs, may hold promise as potential therapeutic agents, which could aid in the development of new treatment targets for bladder cancer; however, further investigation is needed to determine their efficacy and safety.

## 3. Discussion

In this study, we conducted a comprehensive bioinformatics analysis to investigate potential biomarkers and mechanisms related to bladder cancer. By utilizing data from the TCGA database, we identified a total of 519 genes that were upregulated and 1020 genes that were downregulated when compared to normal samples. Among the findings, Polo-like kinase 1 (*PLK1*) emerged as a key factor. *PLK1* plays a significant role in the initiation, maintenance, and completion of mitosis. The malfunction or overexpression of *PLK1* can contribute to cancer development and progression. Elevated levels of *PLK1* have been observed in numerous human cancers and are linked to poorer prognoses in these conditions [19,20]. *AURKB* was found to be highly expressed and linked to prognosis in patients with bladder cancer. Its expression was positively correlated with that of Mitotic arrest deficient 2-like protein 2 (*MAD2L2*) [21]. *AURKB* interacts with and regulates *MAD2L2* expression in bladder cancer cells. Knockdown of *AURKB* resulted in reduced proliferation, migration, invasion, and cell cycle progression in bladder cancer cells. *CCNB1* is implicated in the development of bladder cancer, and its silencing has been shown to slow tumor growth and improve prognosis [22]. Research has shown that *CCNB1* is significantly overexpressed in a range of human tumors and is associated with tumor cell proliferation, metastasis, and poor outcomes [23]. *TOP2A* is found to be upregulated in bladder cancer samples, particularly in high-grade and advanced-stage tumors, as compared to normal epithelial tissue. This upregulation of *TOP2A* is essential for the proliferation, invasion, and survival of BLCA cells [24]. Moreover, *CCNA2*, a cyclin that functions as a regulator of CDKs, is upregulated in bladder cancer [25,26]. Elevated levels of *CCNA2* have been correlated with a poorer prognosis in patients with bladder cancer, indicating that higher expression of this cyclin may be associated with more adverse outcomes and a more aggressive disease course. Following the enrichment analyses using GO and KEGG pathways, it was found that the genes exhibiting upregulated expression were largely associated with several key biological processes. These include the organization of the extracellular matrix, differentiation of endodermal cells, cell adhesion mechanisms, processes of angiogenesis, the assembly of collagen fibrils, and various cell–cell signaling pathways.

The GSVA analysis suggested that varying expression levels of hub genes could impact multiple signaling pathways associated with disease progression. These pathways include PI3K/AKT/mTOR signaling, apoptosis, Wnt/beta-catenin, and Apical Junction. Molecular alterations and elevated activity in the PI3K/AKT/mTOR signaling pathway are commonly observed in cancer [27]. Due to its crucial role in regulating cell growth, survival, and metastasis, components of this pathway are considered promising targets for pharmacological intervention. Various molecular alterations within the PI3K/AKT/mTOR pathway have been identified in BLCA cases. The Wnt signaling pathway, along with its associated genes, proteins, and other molecules, plays a crucial role in various biological processes across different tumors. In bladder cancer, the Wnt signaling pathway has a well-defined regulatory function. Several components linked to this pathway are potential therapeutic targets for treating bladder cancer [28]. Additionally, our investigation revealed associations between these hub genes and the expression of several disease-related genes, such as *MET*, *BRAF*, *PTEN*, *EGFR*, and *PIK3CA*. Our data demonstrate that high levels of *CCNA2*, *CCNB1*, *CDK1*, *PLK1*, *TOP2A*, *AURKA*, *AURKB*, *FOXM1*, and *CALML5* were positively associated with *TP53*, *MET*, *PIK3CA*, *CDH1*, *PALB2*, *KRAS*, *MSH2*, *MSH6*, and *MLH1*. Notably, *MSH2* and *MSH6* demonstrated a high correlation with these genes. *MSH2* and *MSH6* are essential components of the Mismatch Repair (MMR) system, responsible for preserving genomic stability by correcting DNA mismatches that occur during DNA replication. Mutations or deficiencies in these genes are frequently linked to different types of cancer [29,30]. The positive correlation suggests that these hub genes might be functionally related to *MSH2* and *MSH6*. This could imply that they are involved in similar pathways or processes, particularly those related to DNA repair and genomic stability.

Transcription factors (TFs) are essential for controlling gene expression and play a significant role in nearly all physiological processes [31]. These proteins bind to specific sequences on target genes, modulating their expression in a time- and tissue-specific manner. By analyzing the characteristics of validated TF binding sites, we can predict target genes using computational methods [32]. In this study, we developed a gene–TF regulatory network and identified 38 TFs that appear to be significant in bladder cancer. Our findings, derived from the Network Analyst, reveal a complex regulatory network with 10 hub genes. Notably, *FOXC1* was found to regulate six of these hub genes, whereas *CREB1*, *GATA2*, *NFYA*, and *SRF* were found to regulate four hub genes. In summary, these transcription factors likely contribute to bladder cancer through their regulatory effects on genes that control critical processes such as cell proliferation, survival, differentiation, apoptosis, and angiogenesis—processes highly relevant to bladder cancer. For instance, FOXC1 is associated with the epithelial–mesenchymal transition (EMT), which is critical for cancer metastasis [33]. Knocking out the FOXC1 significantly reduces the emergence of drug resistance and cell survival of cisplatin treatment. This suggests that FOXC1 binds to accessible enhancers to promote cisplatin resistance in bladder cancer cells. Targeting FOXC1 could be a promising therapeutic strategy to combat cisplatin resistance and enhance treatment efficacy [33]. CREB1 promotes cell growth and division, which can contribute to tumor progression in various cancers [34]. Thus, the interactions of these TFs with hub genes in the regulatory network highlight their potential as key regulators of bladder cancer biology and as possible targets for therapeutic intervention.

DGIdb v.5.0.7 is a curated database that compiles drug–gene interactions from multiple sources, including DrugBank, PharmGKB, ChEMBL, Drug Target Commons, and others. By utilizing the DGIdb database, we identified a total of 10 drugs associated with each hub gene. Valrubicin is an N-trifluoroacetyl 14-valerate derivative of the anthracycline doxorubicin, recognized for its anti-tumor properties. Pazopanib is an oral, multitargeted tyrosine kinase inhibitor that disrupts tumor angiogenesis and cell proliferation [35]. Paclitaxel, a broad-spectrum anticancer drug, is widely used as a standard chemotherapeutic agent for various cancers [36]. In our study, these drugs were found to interact with *AURKA*, suggesting that it could be a potential drug target for pazopanib and paclitaxel therapy.

In addition, immune checkpoint inhibitors (ICIs) have emerged as a promising therapeutic approach, particularly in the muscle-invasive and metastatic stages of bladder cancer. The key ICIs target the PD-1/PD-L1 and CTLA-4 pathways, which tumors use to evade immune detection. Pembrolizumab, Atezolizumab, Nivolumab, and Durvalumab have been tested against the PD-1/PD-L1 pathway, while Ipilimumab and Tremelimumab target CTLA-4 [37,38]. These inhibitors have demonstrated encouraging results in neoadjuvant and metastatic settings, improving pathological response rates with acceptable safety profiles. Despite the advantages of our study, there are several limitations to consider. One notable limitation is the absence of independent experimental validation. Although we identified four hub genes, their specific roles in bladder cancer pathogenesis have not been extensively validated, and their mechanisms of action are not fully understood. We recognize the importance of rigorous experimental validation for the predicted drug–gene interactions and their effects. Furthermore, while our findings are promising, translating these results into clinical practice will require comprehensive clinical trials to confirm both efficacy and safety.

## 4. Material and Methods

### 4.1. Dataset

The bladder cancer dataset used in this study was retrieved from the TCGA database (https://portal.gdc.cancer.gov/, accessed on 10 June 2024) [18]. To analyze gene expression differences between cancerous and normal tissue samples, we used both the Limma [39] and BIOMAX [40] tools. For gene selection, we applied a threshold where genes with an adjusted *p*-value < 0.05 and a log_2_-fold change >2 were considered significant. Subsequently, the genes identified as upregulated and downregulated based on these thresholds were used in further analysis. This includes pathway analysis and the development of the PPI network.

### 4.2. Functional Enrichment Analysis of the DEGs

The biological functions associated with differentially expressed genes were explored using the DAVID tool [41]. This analysis covered both upregulated and downregulated genes. Gene Ontology (GO) analysis [42] was carried out to categorize the genes based on their functional roles and biological processes. Following this, the Kyoto Encyclopedia of Genes and Genomes (KEGG) pathway analysis [43] was performed to identify the pathways related to these genes. A significance threshold of *p* ≤ 0.001 was applied to determine the statistical significance of the results in both analyses.

### 4.3. Key Regulators (KRs)

The exploration of gene regulatory functions began with the development of a protein–protein interaction (PPI) network using the STRING database v.12.0 [44]. Following this, the network’s topological properties were analyzed using the CytoHubba [45] plugins in Cytoscape [46]. The nodes with the highest degree, indicating the most influential hubs, were identified. Subsequently, the top 10 genes displaying the highest degree of topological characteristics were compared to determine the key regulatory genes.

### 4.4. Molecular Complex Detection (MCODE)

The network clusters were reanalyzed using the Cytoscape plugin application known as MCODE. The analysis was performed using the following parameters: degree cutoff = 2, node score cutoff = 0.2, k-core = 4, and max depth = 100 [47]. Subsequently, the top two modules were selected from the analysis results. Following these, the top ten hub genes were identified using the Cytoscape plugin “cytoHubba”.

### 4.5. Receiver Operating Characteristic (ROC) Curve Analyses

An ROC curve was generated for hub genes using logistic regression analyses [48]. Logistic regression models are advantageous, as they offer straightforward interpretations of coefficients, which facilitates understanding how different predictors impact the outcome, such as biomarker status or disease prognosis. The ROC curve illustrates the true positive rate (sensitivity) on the vertical axis versus the false positive rate on the horizontal axis across various threshold values. Additionally, the AUC (area under the curve) is calculated to quantify the model’s ability to differentiate between classes. An ideal classifier scores an AUC of 1.0, while a model that performs no better than random chance yields an AUC of 0.5. For clarity in the calculations:

$$\mathrm{True}\;\mathrm{positive}\;\mathrm{rate}\;(\mathrm{TPR})=\frac{TP}{TP+FN}$$

where TP represents the true positive and FN represents the false negative.

$$\mathrm{False}\;\mathrm{positive}\;\mathrm{rate}\;(\mathrm{FPR})=\frac{FP}{FP+TN}$$

where FP is the false positive and TN is the true negative.

The AUC is derived by integrating the area under the ROC curve, reflecting the model’s effectiveness in distinguishing between different classes [49].

### 4.6. Gene Set Variation Analysis (GSVA)

The GSVA algorithm [50] was applied to thoroughly score each gene set. This technique works by converting gene-level variations into pathway-level changes. Specifically, it assigns scores to the gene sets of interest, which, in turn, helps in assessing the biological functions of the samples.

### 4.7. Gene–Transcriptional Factor (TF) Regulatory Network

To construct the TF–gene interactions for the hub genes, we used NetworkAnalyst (http://www.networkanalyst.ca/faces/home.xhtml, accessed on 10 July 2024). Initially, the transcription factors (TFs) associated with the hub genes were predicted using the JASPAR database [51]. Subsequently, we constructed and visualized the transcriptional regulatory network using Cytoscape v.3.10.2 [46].

### 4.8. Drug–Gene Interactions

To identify potential target drugs for hub genes, we used the Drug–Gene Interaction Database (DGIdb) [52]. DGIdb is an online resource that provides information on drug–gene interactions and druggable genes. These data are compiled from a variety of sources, including scientific publications, databases, and other web-based sources. The interaction scores were visually represented using bar plots, which were generated with the ggplot2 package in R v.4.4.0 [53].

## 5. Conclusions

This study developed a computational framework to discover biomarker genes for bladder cancer using gene expression profiles from the TCGA database. By employing network biology, we explored biomarkers within the framework of biological networks, including protein–protein interaction networks, metabolic networks, and gene regulatory networks. This approach provides a more comprehensive understanding of how biomarkers interact and function within biological pathways. A total of 10 key regulator genes were discovered in this study. These genes highlight key molecular pathways involved in the disease’s development, progression, and recurrence, suggesting that these key genes may serve as potential biomarkers for managing bladder cancer. We also analyzed the gene–drug and gene–TF interactions. The drugs associated with key genes hold promise for targeted therapies, which could lead to more effective treatments personalized to individual genetic profiles, thereby enhancing patient outcomes. Additionally, our analyses reveal important transcription factors (TFs) that play a crucial role in driving the progression and aggressive characteristics of bladder cancer.

## Data Availability

Enquiries regarding the original contributions presented in the study can be directed to the corresponding author.

## Supplementary Materials

The following supporting information can be downloaded at: https://www.mdpi.com/article/10.3390/ijms252010997/s1.
